# Experimental Investigation of Delamination in Composite Continuous Fiber-Reinforced Plastic Laminates with Elastic Couplings

**DOI:** 10.3390/ma13225146

**Published:** 2020-11-15

**Authors:** Jakub Rzeczkowski, Sylwester Samborski, Marcelo de Moura

**Affiliations:** 1 Department of Fundamentals of Technology, Lublin University of Technology, Nadbystrzycka 38, 20-618 Lublin, Poland; 2 Department of Mechanical Engineering, Lublin University of Technology, Nadbystrzycka 36, 20-618 Lublin, Poland; s.samborski@pollub.pl; 3 Department of Mechanical Engineering and Industrial Management, University of Porto, 4200-465 Porto, Portugal; mfmoura@fe.up.pt

**Keywords:** DCB test, acoustic emission, elastic couplings, delamination, composite laminates

## Abstract

This paper presents an experimental evaluation of influence of the elastic couplings on the fracture toughness as well as on delamination initiation and propagation in carbon/epoxy composite laminates. For this purpose the mode I double cantilever beam (DCB) tests according to the American Society for Testing and Materials (ASTM) D5528 Standard were performed on specimens with different delamination interfaces and specific lay-ups composition exhibiting the bending-twisting (BT) and the bending-extension (BE) couplings. The critical strain energy release rates (mode I c-SERR, *G*_IC_) were calculated by using the classical methods, namely: the modified beam theory (MBT), the compliance calibration (CCM) and the modified compliance calibration (MCC). In order to evaluate an accuracy of the different methods, the values of c-SERR obtained by using standardized data reduction schemes were compared with values calculated by using the compliance based beam method (CBBM). All the methods give rise to comparable values of the *G*_IC_, which makes the CBBM an appealing choice, since it does not depend on crack length monitoring during the test. Initiation and propagation of delamination were investigated by using the acoustic emission (AE) technique. Moreover, the scanning electron microscope (SEM) analysis were performed after the experimental tests in order to investigate a fracture surface at delamination plane.

## 1. Introduction

Carbon fiber reinforced plastic (CFRP) composites made from long and continuous fibers demonstrate excellent properties such as low weight and also lifetime maintenance costs owing to their fatigue and corrosion resistance. Those features make composite laminates widely used in various industry sectors. On the other hand, CFRP laminates are often subjected to different loading conditions, which may cause damage of those materials. The most dangerous form of failure in composites is delamination. The fracture toughness represented by the strain energy release rate (SERR) can be determined in accordance with the American Society for Testing and Materials (ASTM) Standards, namely: for the opening mode I, the double cantilever beam (DCB) [1] test is commonly used, whereas the shearing fracture toughness can be obtained by employing the end-notched flexure (ENF) test [2]. The scope of the DCB test is measurement of the mode I fracture toughness (*G*_IC_) of unidirectional [0°]_n_ laminates according to available standards. However, a multidirectional (MD) laminates with different fibers orientation at the delamination plane are commonly used in most composite structures applications. Many experiments were prepared on the mode I fracture of multidirectional laminates with different delamination interfaces [3,4]. Apart from experimental investigation several authors conducted numerical analysis on delamination problem in the continuous fiber-reinforced laminates [5,6,7,8,9,10,11]. When delamination propagates between different oriented layers, various undesirable phenomena such as mode-mixity and crack jumping to neighbor interface may occur [12]. Other parameters that may influence delamination resistance are: specimen geometry, stacking sequence and intralaminar damage [13]. Although the initiation toughness is often considered the most important in design point of view, there is significant interest in the measurements of propagation toughness values which are represented by the *R*-curve effects [14]. Schön et al. [15] prepared an experimental and numerical investigations of fracture toughness prepared on DCB specimens with different delamination interfaces as well as for different materials. Experimental results revealed, that non-zero interfaces had a plateau value approximately four times higher than the initial value, which was almost the same as for the zero interface. Higher toughness values could be probably caused by initial crack deviation from the original symmetric plane. In reality, anticlastic curvature in DCB specimens caused the highest values of the mode I SERR at the center and the lowest at the specimen edges. This non-uniformity was correlated with a non-dimensional ratios *D*_c_ and *B*_t_ introduced by Davidson et al [16]. Hence, in order to correlate the difference in strain energy release rate at a specimen’s center versus that at its edges a ratio *D*_c_ = *D*_12_/(*D*_11_*D*_12_) was used, whereas a stiffness ratio *B*_t_ = *D*_16_/*D*_11_, was found to correlate with the asymmetry in the SERR distribution about the center of the specimen’s width. In order to neglect the non-uniformity of *G*_I_ in determination of *G*_IC_ it was proposed that the *D*_c_ parameter should be less than 0.25. Nevertheless, Olsson [17] showed that the specimen was in a state of plane strain for small values of *a*_0_/*b* and globally was in a state of a plane stress for large aspect ratios. It was also revealed, that when specimens transferred from plane stress to plane strain the average value of the mode I fracture toughness decreased. Hence, at parameters of DCB specimen such as thickness (*h*), initial delamination length (*a*_0_) or width (*b*) significantly affected the *G*_IC_ value. Therefore, Shokrieh [18] introduced a non-uniform ratio *β* = (*G*_Imax_ − *G*_Iavg_)/*G*_Iavg_%, which take into account the influence of abovementioned factors on the SERR distribution along width of the DCB specimen. In general *β* was a function of non-dimensional ratio *D*_c_ and geometrical ratios *a*_0_/*b* and *a*_0_/*h*, *β* = *f*(*D*_c_, *a*_0_/*b*, *a*_0_/*h*). Results showed that the mode I fracture toughness of MD double cantilever beam specimens with 0°//0° fiber angle at delamination plane and *β* < 20% could be predictably measuring the *G*_IC_ of the unidirectional UD plies with an error lest than 10%. Moreover, it was proved that *β* ratio had significant influence on the initiation delamination resistance and it was necessary to be considered in calculation of *G*_IC_ by analytical relations which based on the beam theories. In practice, determination of the fracture toughness in composites may be difficult when elastic couplings occur in laminate [19]. For example, this is the case for laminates where the bending-twisting or the bending-extension couplings take place. Previous research prepared by the author of this paper [20] conducted on the DCB specimens exhibited elastic couplings under the mode I DCB test revealed, that non-zero components in the bending stiffness matrix [B] caused couplings between the bending moment and the twisting curvature as well as between the twisting moment and the mid-plane normal strains. Presence of shearing deformation and twisting curvature caused non-uniform delamination front which in results could produce an inaccuracies in the fracture toughness determination. In order to investigate the influence of irregular crack front on delamination initiation and the mode I strain energy release rate distributions Jiang et al. [21] prepared a modified DCB specimen. They concluded, that when the effects of visual deviation *λ* and the non-uniformity coefficient *β* were not taken into account the fracture toughness values obtained by using classical methods based on the beam theory were underestimated. Moreover, when the effect of curved delamination front during experimental measurement was neglected the errors in the mode I SERR values obtained for multidirectional DCB specimens could reached level up to 47.6% [22]. Another problem that can generate errors in measurements of the initiation and propagation values of SERR is visual observation of crack length through the test. Actually, the fracture process zone (FPZ) which develops ahead of the crack tip in result of micro-cracking and plasticity can cause unreliable monitoring of crack position during the mode I tests [23]. Abovementioned factors are not included in classical calculation schemes of the c-SERR recommended by the ASTM Standard. Moreover, visual detection of crack length are incredibly difficult to perform experimentally. To overcome this difficulties, an author of this paper proposed the compliance-based beam method (CBBM) [24]. This methodology is based on the crack equivalent concept and depends only on the specimens compliance during the test. In addition, this method do not require measurements of actual crack length during propagation which can produces non-negligible errors in the fracture toughness measurements [25,26]. Moreover, this methodology can be a great tool to create an artificial *R*-curve, which was proved in [27] where an excellent agreement between the experimental and numerical *R*-curves was achieved demonstrating adequacy of the proposed method.

Determination of crack propagation in structures plays an important role in the fracture mechanics of composites and can be investigated by using the acoustic emission (AE) technique [28]. The non-destructive AE method can be utilized in detecting the real delamination onset in damage initiation analysis. Nikbakht et al. [29] focused on damage analysis of the glass/epoxy multidirectional double cantilever beam specimens. Obtained AE data revealed that delamination initiated before any visual method can even see micro cracks. Saeedifar et al. [30] also used AE method during the mode I DCB tests prepared on glass/epoxy composite specimens to determine of crack tip position and to evaluate the interlaminar delamination resistance. They concluded, that obtained outcomes were similar to results calculated by using the nonlinearity (NL) method recommended in ASTM Standard. Generally, the acoustic emission methodology is a powerful technique to examine the initiation and propagation of delamination in composite laminates.

The main goal of this paper is to evaluate the influence of elastic couplings on behavior of the carbon/epoxy composite laminates subjected to the DCB tests. Specimens with different delamination interfaces and stacking sequences exhibited the bending-twisting and the bending-extension couplings were examined. Values of the critical strain energy release rate obtained by using standardized methods were compared with results obtained by using the CBBM method. In addition, experimental investigations of initiation and propagation of delamination were executed by using the acoustic emission technique and the scanning electron microscopy.

## 2. Elastic Coupling Phenomenon in Composites

The coupling phenomenon is an intrinsic attribute of the layered CFRP structures with different orientations of reinforcing fibers in subsequent plies when the lay-ups are non-symmetric. The constitutive equations describing all possible couplings in a laminate can be written in a block-matrix form [31]:

(1) $$\left\{\begin{array}{l} N\\ M \end{array}\right\}=\left[\begin{array}{cc} A & B\\ B & D \end{array}\right]\left\{\begin{array}{c} \varepsilon^{0}\\ \kappa^{0} \end{array}\right\}$$

where the [N] and the [M] matrices stand for the laminate’s forces and moments, respectively. The last column matrix depicts deformation of the neutral plane of the composite (ε^0^—strains, κ^0^—curvatures). The key element in Equation (1) is the block matrix in the square brackets; its constituents are the extensional stiffness matrix [A], the coupling stiffness matrix [B] and the bending stiffness matrix [D]. The influence of elastic coupling phenomena on behavior of CFRP laminates subjected to the mode I DCB test were analytically analyzed in previous paper [20].

## 3. Experimental Procedures

### 3.1. DCB Specimens

**Figure 1:**
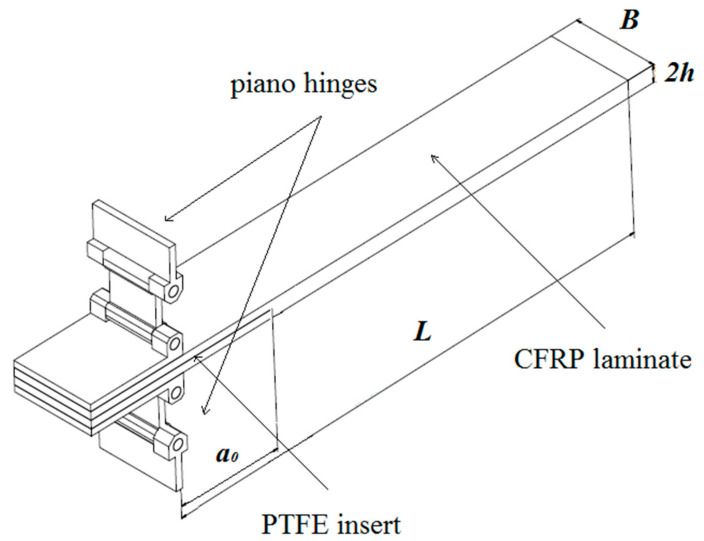
Geometry of the DCB specimen.

Experimental DCB specimens were performed on uniform thickness multidirectional (MD) carbon/epoxy composite laminates with different delamination interfaces: [0°_19_//0°_19_], [0°_18_/*θ*//-*θ*/0°_18_], [0°_18_/*θ*//*θ*/0°_18_] and [0°_18_/0//*θ*/0°_18_]. The fiber orientation angle *θ* was {30°, 45°, 60°, 90°}. Additionally, the bending-twisting (BT) and the bending-extension (BE) coupled laminates with specific stacking sequences which were presented in Table 1 were examined. In order to initiate delamination a 12 µm thick teflon PTFE foil was placed at mid-thickness of the laminate. In addition, both edges of specimens were coated white paint to increase accuracy of visual detection of crack tip. Specimen with the geometry: width *B* =25 mm, total thickness 2*h* = 4.98 mm and total length *L* =175 mm was presented in Figure 1. The loading piano hinges according to the Standard ASTM D5528 were adhesively bonded to the specimen allowing to apply crack opening force. Material properties of tested laminates were obtained during tensile tests and were summarized in Table 2.

### 3.2. Test Procedure

The double cantilever beam (DCB) tests were performed on Shimadzu ASG-X (Lublin, Poland) tensile testing machine (equipped with 1kN load cell) in accordance with the ASTM D5528 Standard procedure with a constant crosshead speed equal 1 mm/min. During all tests the load-displacement data were recorded by the Trapezium-X software. In addition, delamination onset as well as all propagation of delamination values were visually observed and marked on both specimen edges which were coated with a white paint. In order to ensure high precision of detecting the very beginning initiation of delamination - and in consequence to obtain high accuracy of the initial *G*_IC_ determination, all experiments were supported by using acoustic emission (AE) technique. The piezoelectric (Fujicera 1045S) AE sensor was adhesively bonded at the top of laminates in 25 mm distance from specimen’s end edge. Elastic waves of the AE signal were recorded continuously by using AMSY-5 Acoustic Emission acquisition system at 10 MHz sampling rate. During all experiments the energy of elastic waves as well as the number of events were registered. The first increase of cumulative counts of the acoustic emission signal was taken into account as initiation of delamination and was called the AE point; note that this point was taken into account in calculations of the mode I critical strain energy release rates. In addition, the real delamination surfaces of the DCB specimens were investigated by using the scanning electron microscope (SEM) (Quanta FEG 450, Pisa, Italy).

### 3.3. Data Reduction Schemes

#### 3.3.1. Classical Methods

Three classical data reduction schemes of calculation of the mode I critical strain energy release rate (c-SERR) is being proposed by the ASTM D5528 Standard, namely: the modified beam theory (MBT), the compliance calibration method (CCM) and the modified compliance calibration (MCC).

The MBT method uses an experimentally determined correction parameter Δ which is generating as a least squares plot of the cube root of compliance versus delamination length *a*. Subsequent parameters are: *B* specimen width, *P* load and *δ* displacement. The mode I interlaminar fracture toughness can be determined with the following equation:

(2) $$G_{\mathrm{IC}}=\frac{3P\delta }{2B(a+\left|\Delta \right|)}$$

The CCM method uses an additional experimentally obtained parameter *n* which is created as the slope of the *ln*(*C*) versus *ln*(*a*). Then, the mode I critical strain energy release rate is calculated as follows:

(3) $$G_{\mathrm{IC}}=\frac{nP\delta }{2aB}$$

In the MCC method the critical SERR can be calculated by using the following equation in which the *A*_1_ is the slope of a least squares plot of delamination length normalized by the specimen thickness *a*/*h* as a function of the cubic root of compliance:

(4) $$G_{\mathrm{IC}}=\frac{3P^{2}C^{2/3}}{2A_{1}Bh}$$

#### 3.3.2. Compliance-Based Beam Method (CBBM)

The critical strain energy release rates calculated by using previous methods (CCM, MBT, MCC) are depended on precise visual crack length measurements during the tests which is not easy to perform experimentally. Moreover, a fracture process zone which develops ahead of crack tip in a results of plastification or micro-cracking makes the visual crack tip localization more difficult. The compliance-based beam method (CBBM) which based on the crack equivalent concept takes into account these features and is described below. This methodology depends only on the specimen’s compliance (*C* = *δ*/*P*) which is expressed as follows:

(5) $$C=\frac{8a^{3}}{E_{1}Bh^{3}}$$

where *a* specimen crack length, *E*_1_ longitudinal elastic modulus, *B* specimen width and *h* is arm’s height. Root rotation effect at crack tip is also taken into account by introduction a correction Δ calculated based on formula proposed by Hashemi et al. [32]:

(6) $$\Delta =h\sqrt{\frac{E_{1}}{11G_{12}}\left[3-2{\left(\frac{\Gamma }{1+\Gamma }\right)}^{2}\right]};\Gamma =1.18\frac{\sqrt{E_{1}E_{2}}}{G_{12}}$$

Here *E*_1_, *E*_2,_ *G*_12_ are material properties of carbon/epoxy laminate. Additionally, in order to account for scatter of elastic properties between different specimens a corrected flexural modulus can be calculated by using Equation (7) where *C*_0_ and *a*_0_ stands for initial compliance and crack length, respectively.

(7) $$E_{f}=\frac{8{\left(a_{0}+\left|\Delta \right|\right)}^{3}}{Bh^{3}C_{0}}$$

The equivalent crack length *a*_e_ (Equation (8)) is a function of the current compliance *C,* which is calculated directly from the measured load-displacement (*P*-*δ*) curve and by using the corrected flexural modulus *E*_f_

(8) $$a_{e}={\left(\frac{CE_{f}Bh^{3}}{8}\right)}^{1/3}$$

The values of the mode I critical strain energy release rate can be calculated from the Irwin-Kies equation:

(9) $$G_{I}=\frac{P^{2}}{2B}\frac{dC}{da}$$

which leads to

(10) $$G_{\mathrm{IC}}=\frac{12P^{2}a_{e}{}^{2}}{B^{2}h^{3}E_{f}}$$

## 4. Effect of Elastic Couplings on Composite Laminates

Coupling behavior is dependent on the form of each of the elements in the extensional [A], the coupling [B] and the bending [D] stiffness matrices. In order to validate the influence of elastic couplings on the DCB laminates, values of the *A*_ij_, *B*_ij_ and *D*_ij_ components of the stiffness matrices and the non-dimensional parameters *D*_c_ and *B*_t_ [9] were calculated for chosen specimens according to the classical lamination theory (CLT) by using MatLab software environment. Obtained values were collected in Table 3 and Table 4. Experimental investigation of influence of the elastic couplings on delamination onset and propagation was presented in the subsequent section.

Composite laminates exhibit coupling between the in-plane shear strains and the extension when *A*_16_ and *A*_26_≠0, and between the bending moments and the twisting curvatures when *D*_16_ and *D*_26_≠0. For the 0°//0° laminate those conditions were equal zero, on contrary to the angle-ply laminates where the extensional stiffness matrix was fully coupled which initiated coupling between the normal stress and the shear strains (in-plane normal forces produced mid-plane shear strains). Considering the coupling stiffness matrix, the *B*_11_, *B*_12_, *B*_22_ and *B*_66_ components were also different from zero which induced shearing deformations under the bending load. Nevertheless, all terms in the coupling matrices [B] were incredibly small. On the other hand, fully coupled bending stiffness matrix [D] with non-zero *D*_16_ component responsible for twisting curvature could cause that specimen subjected to the mode I DCB test tried to twist itself around it’s central axis. In addition, for all tested specimens with different delamination interfaces, the non-dimensional *D*_c_ parameter was close to zero and the bending-twisting coupling intensity parameter *B*_t_ was equal zero. It means that influence of elastic couplings on the strain energy release rate was insignificant in this case. For the laminates with specific stacking sequences where the bending-twisting and the bending-extension couplings occurred, the values of the non-dimensional parameters were much greater and reached: *D*_c_ = 0.2384 and *B*_t_ = 0.2363 for the BT specimen as well as *D*_c_ = 0.4089 and *B*_t_ = 0 for the BE specimen. In this case the elastic coupling phenomena strongly influenced on behavior of the laminates and in consequence the SERR distribution along specimen width which was proved by an author of this paper in [33,34].

**Figure 2:**
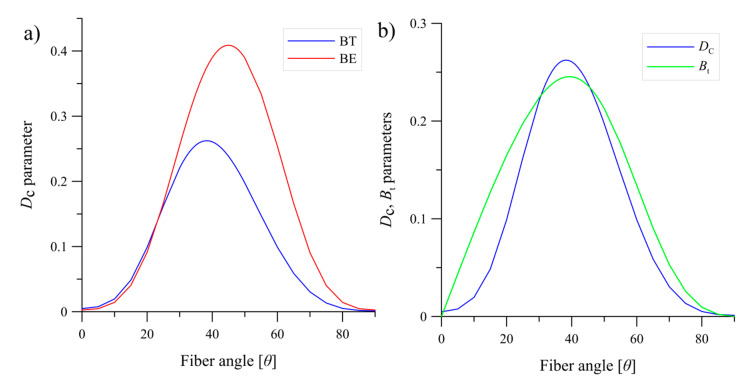
Non-dimensional parameters: (**a**) values of the *D*_C_ parameter calculated for different fiber angles *θ* for the bending-twisting and the bending-extension laminates, (**b**) values of the *D*_C_ and *B*_t_ parameters calculated for different fiber angles *θ* for the bending-twisting laminate.

Values of the *D*_c_ and *B*_t_ parameters depended not only on the specific laminate configuration but also on the fiber *θ* angles which was presented in Figure 2. For fiber angles less than *θ* = 20° and greater than *θ* = 60° values of the non-dimensional parameter *D*_c_ were small. The greatest values were obtained for fiber angles range between *θ =* 35° and *θ* = 45° reaching maximum for the bending-twisting laminate equal *D*_c_ = 0.2623 for *θ* = 38° and for the bending-extension laminate *D*_c_ = 0.4089 for *θ* = 45°. For the BT specimen values of the non-dimensional parameter *B*_t_ were similar to *D*_c_ whereas for the BE laminate those values were equal zero for the entire range of fiber angles.

## 5. Experimental Results

### 5.1. Critical Strain Energy Release Rate (c-SERR)

Table 5 presents the results of calculation of the mode I critical strain energy release rate obtained for specimens with different delamination interfaces as well as for the laminates exhibiting elastic couplings. Values of the critical strain energy release rates calculated by using the CBBM method were discussed below. For the angle-ply laminates maximum value of the *G*_IC_ was obtained for specimen with interface 60°//60° and was equal to 0.79 N/mm. Slightly less value of the critical strain energy release rate was obtained for the 0°//60° interface (c-SERR = 0.74 N/mm). Specimens with delamination interfaces 30°//−30° and 90°//90° exhibited values of the *G*_IC_ on average level equal 0.58 N/mm. Considering 0°//45°, 0°//90° and 60°//−60° interfaces, the mode I strain energy release rates were equal to *G*_IC_ = 0.39 N/mm, *G*_IC_ = 0.45 N/mm and *G*_IC_ = 0.45 N/mm, respectively. The lowest value of the mode I c-SERR was obtained for the unidirectional laminate and was equal to 0.15 N/mm. For the interface 45°//45° the critical strain energy release rate was greater and equal 0.18 N/mm. For specimens with delamination interface 0°//*θ* and *θ//θ* (without interface 0°//90°) with the growth of *θ* angle the values of mode I c-SERR also increased. Comparing the coupled composite laminates the greatest value of the mode I c-SERR was reached for specimen with the bending-extension coupling which was equal 0.65 N/mm. For specimen with the bending-twisting coupling the mode I fracture toughness was about two times smaller and equal 0.38 N/mm. Values of the critical strain energy release rate obtained by using methods recommended by the ASTM Standard were on similar level. Moreover, differences between the standardized data reduction schemes and the compliance based beam method were small. However, for all tested specimens values of the *G*_IC_ obtained by using the CBBM were slightly greater. Nevertheless, those discrepancies between the standardized and non-standardized methods were not significant. In addition, transformation from the plane strain state to the plane stress state with increasing values of the *a*_0_/*b* ratio described in [10] had no major effect on the c-SERR values.

**Figure 3:**
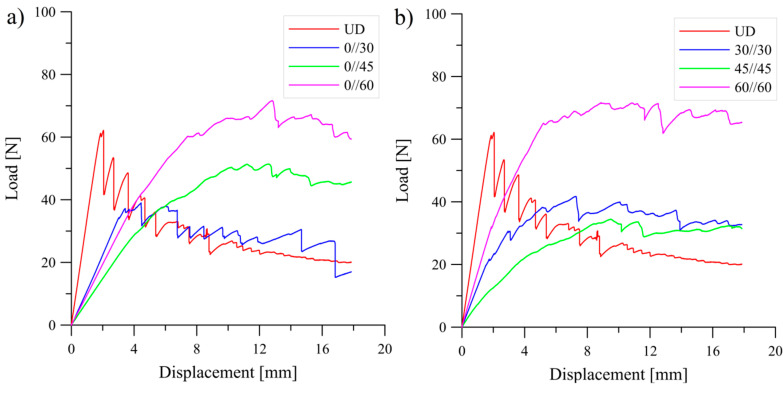
Load-displacement curves obtained during the DCB experiments: (**a**) for the UD and the 0°//*θ* laminates, (**b**) for the UD and the *θ*//*θ* laminates.

**Figure 4:**
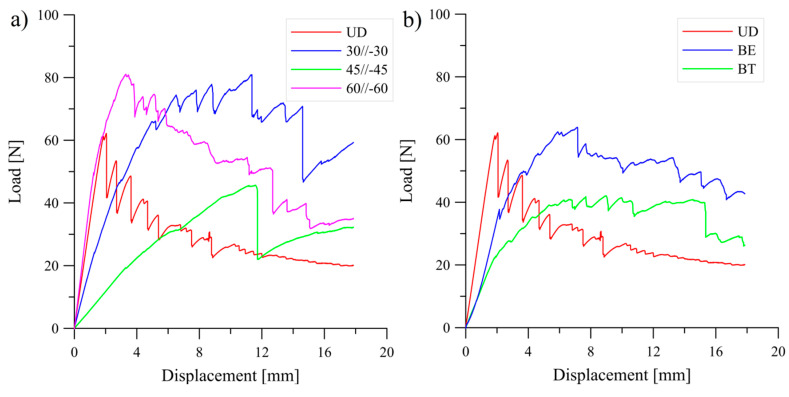
Load-displacement curve obtained during the DCB experiments: (**a**) for the UD and the *θ*//−*θ* laminates, (**b**) for the UD and the coupled laminates.

Typical load versus displacement curves which were obtained during experimental tests were presented in Figure 3 and Figure 4.

### 5.2. Analysis of Delamination Initiation and Propagation

**Figure 5:**
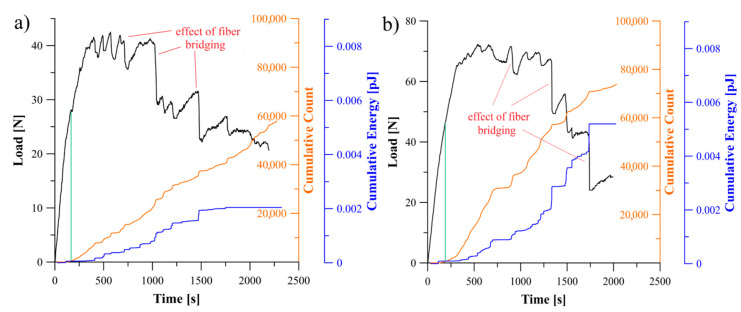
Relation between load, cumulative count and energy of AE signal obtained during the DCB experiment: (**a**) for specimen exhibiting the bending-twisting elastic coupling, (**b**) for specimen with 60°//60° delamination interface.

**Figure 6:**
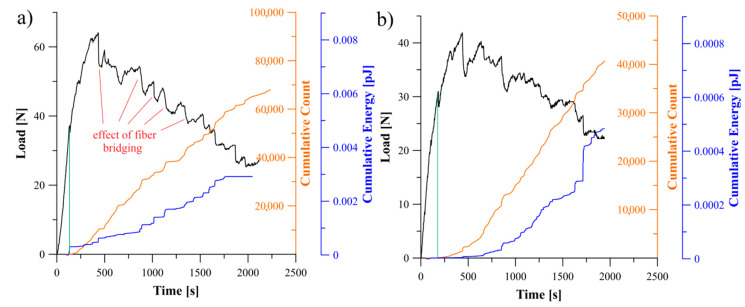
Relation between load, cumulative count and energy of AE signal obtained during the DCB experiment: (**a**) for specimen exhibiting the bending-extension elastic coupling, (**b**) for specimen with 30°//30° delamination interface.

**Figure 7:**
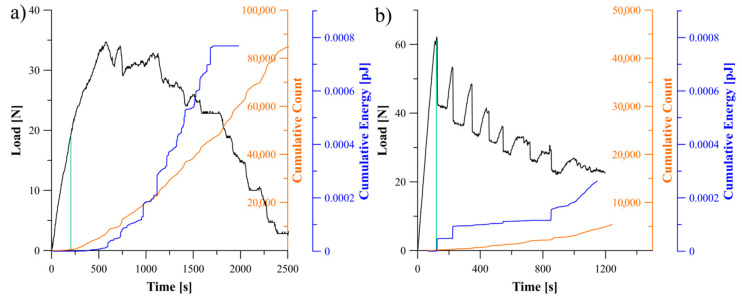
Relation between load, cumulative count and energy of AE signal obtained during the DCB experiment: (**a**) for specimen with 45°//45° delamination interface, (**b**) for UD laminate.

The acoustic emission behavior during propagation of delamination process could be divided into three regions, namely: first region, where any damage mechanism was not occurred in laminate which in consequence no significant AE activity could be observed, second region where delamination initiation caused abrupt acoustic emission activity and third region with different AE activities caused by propagation of delamination. In Figure 5, Figure 6 and Figure 7 relations of the load versus the cumulative AE energy and counts for the laminates with 30°//30°, 45°//45°, 60°//60° delamination interfaces as well as for the BT and the BE specimens were presented.

Considering specific period of time, it could be observed, that for all tested specimens sudden changes in load led in a similar increase in the acoustic emission rate. This could be caused of by an increase in the rate of AE events occurred in the corresponding range of time. Moreover, the rate of changes and the trend in cumulative AE counts were similar to those of cumulative acoustic energy in every particular range of time. It proved that more AE energy emission caused more events. Although some similarities in general behavior of the counts and acoustic emission energy could be observed, however, there were some detailed differences for each laminates, which could be caused by the effect of delamination interface as well as by the specific sequence of laminate layers.

**Figure 8:**
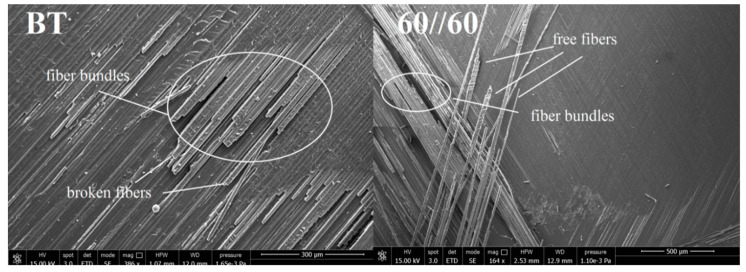
Delamination surfaces after the scanning electron microscope (SEM) investigations for the specimen with 60°//60° delamination interface and for the bending-twisting (BT) laminate.

In the case of the bending-twisting coupled laminate (Figure 5a) the AE graphs experienced a number of sharp jump. Similar trend of the load and AE curve during propagation could be observed also for the laminate with 60°//60° interface (Figure 5b). This might be due to fiber bridging phenomena. The differently oriented fibers at the interface might bridge and then their abrupt debonding or break could produce sudden occurrence of large amount of AE emission and many events which resulted in violent crack propagation observed during the DCB experiment. In some cases, single bridging fibers were merged into bridged bundle which were obstacle for micro-cracks propagation. The abrupt released of that bundle resulted in acoustic energy jump caused by tremendous AE events at crack front occurred in that time. This phenomenon highlighted in Figure 5a, b. Microscopic SEM investigation of delamination surface after the DCB test revealed, that both for the BT and the 60°//60° specimen such bridged bundle occurred which was presented in Figure 8. The nature of fiber bridging depended on the fiber angle at the interface. For the 60°//60° interface bridged fibers released with greater AE energy than specimens with 30°//30° and 45°//45° interfaces.

**Figure 9:**
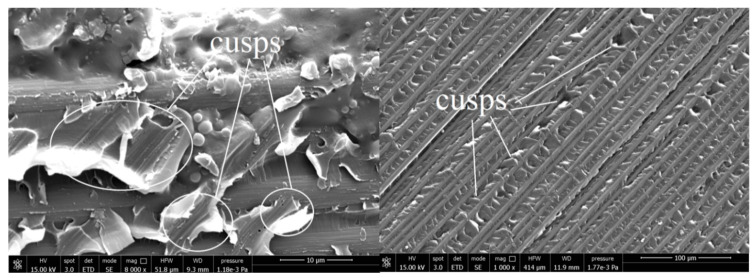
Delamination surfaces after the SEM investigations of the bending-extension specimen.

For the BE specimen (Figure 6a) the load and the AE energy jumped very frequently, however, each jump emitted less energy relative to the bending-twisting laminate. It could be caused that releasing of acoustic emission energy and the cycles of bundle bridging occurred in a shorter period of time and consequently less events happened and less AE energy was emitted. Similar situation could be observed for the angle-ply laminates with delamination interfaces 30°//30° and 45°//45°, which was presented in Figure 6b and Figure 7a. In addition, the SEM analysis revealed that for the BE specimen a non-zero value of the *B*_11_ term caused that the stress field was strongly dominated by the mode II component. As the shear component increased the toughness also increased which was related to the formation of cusps (Figure 9). In macro-scale this occurred as an surface roughness increase. Microscopically cusps were formed by the development of angled cracks, that with increasing of the mode II component extended towards the plies and coalesced. Locally, cusps could cause a cleavage fracture which led to increase in absorption of fracture energy and in consequence increase in the fracture toughness. It was confirmed during determination of the c-SERR for the bending-extension specimen for which the non-zero *B*_11_ component of coupling stiffness matrix is responsible for the shearing effect under the bending load. Results of calculations revealed that the value of the mode I critical strain energy release rate for the BE laminate was greater than for the BT specimen for which the *B*_11_ component was close to zero. Moreover, microscopic SEM investigation of the BE specimen showed a number of cusps spread on delamination plane.

**Figure 10:**
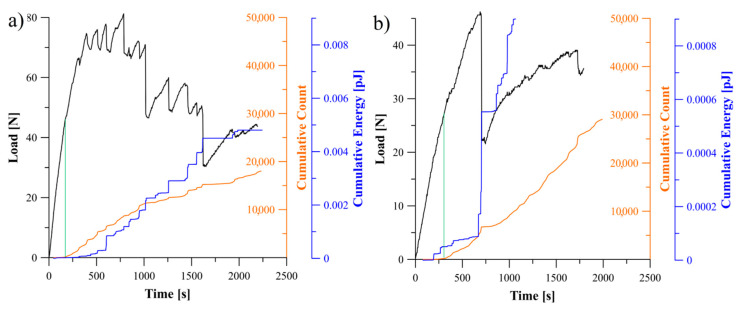
Relation between load, cumulative count and energy of AE signal obtained during the DCB experiment: (**a**) for specimen with 30°//−30° delamination interface, (**b**) for specimen with 45°//−45° delamination interface.

**Figure 11:**
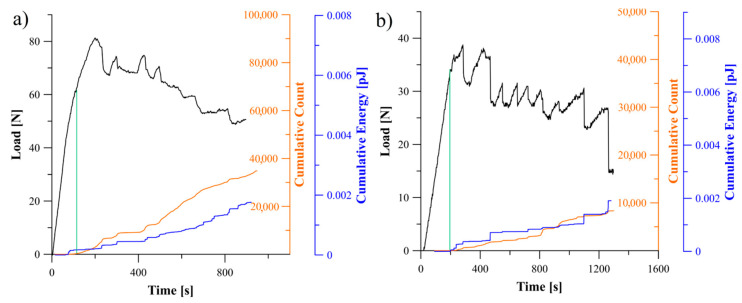
Relation between load, cumulative count and energy of AE signal obtained during the DCB experiment: (**a**) for specimen with 60°//−60° delamination interface, (**b**) for specimen with 0°//30° delamination interface.

**Figure 12:**
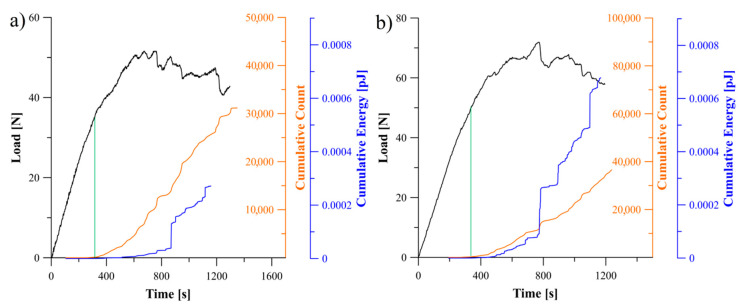
Relation between load, cumulative count and energy of AE signal obtained during the DCB experiment: (**a**) for specimen with 0°//45° delamination interface, (**b**) for specimen with 0°//60° delamination interface.

Relations between the load, cumulative count and energy of AE signal obtained for the specimens with *θ*//−*θ* and 0°//*θ* delamination interfaces were presented in Figure 10, Figure 11 and Figure 12. The greatest values of released AE energy during delamination process were observed for the laminates with interfaces 30°//−30°, 60°//−60° and 0°//30°. For the 45°//−45° specimen a characteristic energy jump caused by sudden propagation of delamination was observed. In the case of the 0°//45° and 0°//60° specimens, a rapid growth of the AE energy occurred. In addition, it was observed that for several tested specimens the AE energy reached established level at the end of propagation whereas the cumulative counts were continuously growing. It could be cause by a steady-state crack propagation connected with occurrence of the matrix cracking with constant rate, which is considered as a major source of acoustic energy. During propagation, such phenomena as fiber-matrix debonding or fiber breakage also generated the acoustic waves but they had less influence on total counts of the cumulative events.

Apart from investigation of delamination propagation, the AE technique could be successfully utilized as non-destructive method in detecting crack of initiation, which played extremely important role in determination of the fracture toughness. In order to obtain the critical strain energy release rate it was necessarily to identify the point on the load versus displacement plot where crack started to propagate. Acoustic emission is a natural phenomenon caused by propagation of transient elastic wave resulted from abrupt energy releasing inside the material. The point called AE was the first sharp increase in the cumulative of the acoustic energy counts. For example, for the BE specimen the first visually observed crack growth was registered when load reached 65 N. It could be caused by phenomenon of bridged bundles of fibers at the crack tip which resisted against a crack growth. However, stored strain energy in specimen (*G*_I_) achieved the critical value (*G*_IC_) when load reached 38 N. Hence, this point should be considered as delamination initiation. It was confirmed by increase in the cumulative counts of acoustic emission signal which corresponded to increase of the cumulative AE energy. Delamination onset (AE point) determined as first increase of acoustic signal was pointed out on all plots as the end of green line.

Proper recognizing of initiation point is often questionable, so the ASTM Standard recommended three different criteria: visually observed crack front (VIS), increase of compliance (5%) and deviation from linearity (NL). Nevertheless, initiation of delamination at interface with different fibers orientation is different from when the delamination initiates between unidirectional plies. Although, the acoustic emission signal for the specimens with 45°//45° and 60°//60° interfaces indicated early damage initiation a crack tip was not visible. In fact, the early micro-cracking in 45° and 60° interfaces created more extensive fracture process zone ahead of the crack tip because of small crack opening displacement. On the other hand, for the specimen with interface 30°//30° as well as for the BT and BE laminate because of greater crack opening displacement caused by existence of larger local normal strains the crack tip was visible. It was correlated with sharp jump on the load curve obtained during the experiment. In this case, evolution of damage along the fibers created a smaller FPZ and much more visible crack onset. Also, the component *D*_16_ might influence on crack tip visibility. Non-zero *D*_16_ term caused that specimen were tensioned on one side and compressed on another which resulted that crack tip was visible only on one edge of tested specimen.

## 6. Conclusions

An experimental study was conducted on the mode I double cantilever beam carbon/epoxy composite beams exhibited different delamination interfaces and elastic couplings. The mode I critical strain energy release rates were calculated by using the standardized data reduction schemes recommended by the ASTM D5528 Standard and the compliance-based beam method which based on the crack-equivalent concept. Results showed that the mode I fracture toughness depended on fiber angle at the interface and type of elastic coupling. Moreover, discrepancies between the *G*_IC_ values obtained by using four different calculation methods were relatively small. Application of the CBBM method to specimens with different delamination interfaces and specific stacking sequences turned out to be advantageous relative to the classical ones since it did not required measurements of crack length during test and takes into account the energy dissipated at the fracture process zone.

Analytical analysis showed that the values of the non-dimensional parameter *D*_c_ and *B*_t_ depended not only on the lay-ups composition but also on fiber angles in specific stacking sequences. The greatest values of the *D*_c_ were obtained for fiber angles range between *θ* = 35° and *θ* = 45° for the bending-twisting as well as for the bending-extension specimens. For the BT laminate values of *B*_t_ parameter were similar to *D*_c_ whereas for the BE specimen, values of coupling intensity parameter were equal zero for entire angles set.

The AE technique was successfully utilized in determination of delamination initiation. The first increase of cumulative counts was considered as a delamination onset.

Acoustic emission investigations and SEM observations revealed that delamination initiation and propagation depend on type of elastic coupling. In the case of the BT and 60°//60° laminates the twisting curvatures caused that fibers merged into bundles and energy released was significantly large. On the other hand, for the 30°//30° and 45°//45° specimens released AE energy was smaller. This confirmed that bridging phenomena also depended on the interface type. Moreover, for the bending-extension specimen, the *B*_11_ term of the coupling stiffness matrix caused contribution of the mode II shearing components in the form of cusps which was proved during the microscopic observations. Presence of the shearing effects could increase total value of the strain energy release rate measured during the mode I DCB test. Moreover, elastic couplings influenced on laminates behavior and decreased visibility of crack tip at one side of specimen during experimental tests.

## Figures and Tables

**Table 1:** Laminate configurations.

Laminate	Stacking Sequence (for One Sub-laminate)
**BT**	(45°/0°/45°/45°/0°/−45°/0°/−45°/−45°/−45°/−45°/0°/−45°/45°/0°/0°/45°/45°)
**BE**	(45°/−45°/0°/−45°/0°/45°/90°/45°/−45°)

**Table 2:** Mechanical properties of carbon/epoxy laminate.

*E*_1_ (GPa)	*E*_2_ (GPa)	*ν*_12_ (-)	*G*_12_ (GPa)
112.105	7.421	0.270	3.338

**Table 3:** Values of the stiffness matrices components calculated for the laminates with different delamination interfaces.

Interface	A (MPa × mm) × 10^5^			B (MPa × mm^2^) × 10^−10^			D (MPa × mm^3^) × 10^5^			*D_c_*	*B_t_*
0°//0°	5.0174	0.0897	0	−0.2910	0.0034	0	8.2946	0.1483	0	0.0048	0
	0.0897	0.3321	0	0.0034	0.0045	0	0.1483	0.5491	0		
	0	0	0.1487	0	0	−0.0011	0	0	0.2458		
30°//30°	4.8980	0.1401	0.0888	−0.2910	0.0034	0	8.2939	0.1485	0.0005	0.0048	0
	0.1401	0.3506	0.0305	0.0034	0.0045	0	0.1485	0.5492	0.0002		
	0.0888	0.0305	0.1991	0	0	−0.0011	0.0005	0.0002	0.2461		
45°//45°	4.8123	0.1570	0.0689	−0.2910	0.0034	0	8.2934	0.1486	0.0004	0.0048	0
	0.1570	0.4026	0.0689	0.0034	0.0045	0	0.1486	0.5495	0.0004		
	0.0689	0.0689	0.2160	0	0	0.0011	0.0004	0.0004	0.2462		
60°//60°	4.7602	0.1401	0.0305	−0.2910	0.0034	0	8.2931	0.1485	0.0002	0.0048	0
	0.1401	0.4884	0.0888	0.0034	0.0045	0	0.1485	0.5500	0.0005		
	0.0305	0.0888	0.1991	0	0	−0.0011	0.0002	0.0005	0.2461		

**Table 4:** Values of the stiffness matrices components calculated for laminates with the bending-twisting (BT) and the bending-extension (BE) couplings.

Laminate	A (MPa × mm) × 10^5^			B (MPa × mm^2^) × 10^−11^			D (MPa × mm^3^) × 10^4^			*D_c_*	*B_t_*
BT	1.4257	0.4512	0	0.0909	0	0.0909	6.6061	2.0906	1.5608	0.2384	0.2363
	0.4512	0.5989	0	0	−0.0909	0	2.0906	2.7751	1.5608		
	0	0	0.4824	0.0909	0	0.1819	1.5608	1.5608	2.2353		
**Laminate**	**A (MPa × mm) × 10^4^**			**B (MPa × mm^2^) × 10^3^**			**D (MPa × mm^3^) × 10^3^**			***D_c_***	***B_t_***
BE	5.7506	2.2560	0	−3.6104	0	0	5.1803	3.3060	0	0.4089	0
	2.2560	4.3726	0	0	3.6104	0	3.3060	5.1606	0		
	0	0	2.4121	0	0	0	0	0	3.4870		

**Table 5:** Critical strain energy release rate (c-SERR) obtained for different DCB specimens by using different calculation methods (average values and standard deviations).

Interface	CCM		MBT		MCC		CBBM	
	*G*_IC_ (N/mm)	Error (%)	*G*_IC_ (N/mm)	Error (%)	*G*_IC_ (N/mm)	Error (%)	*G*_IC_ (N/mm)	Error (%)
0°//0°	0.14	0.08	0.14	0.17	0.14	2.08	0.15	0.26
0°//30°	0.18	2.50	0.19	2.61	0.19	1.95	0.21	6.28
0°//45°	0.39	3.49	0.38	4.07	0.38	4.68	0.39	2.21
0°//60°	0.53	4.33	0.51	1.16	0.50	1.93	0.74	10.08
0°//90°	0.40	10.28	0.42	13.40	0.40	10.55	0.45	12.29
30°//30°	0.16	1.68	0.17	2.25	0.17	2.11	0.17	2.37
30°//−30°	0.49	11.54	0.5	11.92	0.51	11.04	0.56	16.66
45°//45°	0.16	1.59	0.16	1.44	0.16	1.36	0.18	0.76
45°//−45°	0.22	7.32	0.22	8.64	0.23	8.76	0.24	8.94
60°//60°	0.67	3.10	0.66	4.11	0.63	5.26	0.79	4.64
60°//−60°	0.46	4.21	0.46	4.87	0.42	3.59	0.45	3.75
90°//90°	0.54	2.20	0.54	3.60	0.54	6.83	0.61	9.33
BE	0.51	3.56	0.50	2.32	0.49	5.38	0.65	9.06
BT	0.36	3.06	0.36	2.59	0.34	2.11	0.38	1.47

## Nomenclature

*a*	crack length
*a*_eq_	equivalent crack length
*a*_0_	initial crack length
*h*	half thickness of specimen
*n*	correction parameter
A	extensional stiffness matrix
*A*_ij_	component of the extensional stiffness matrix
*A*_1_	correction parameter
B	coupling stiffness matrix
*B*	specimen width
*B_ij_*	component of the coupling stiffness matrix
*B_t_*	non-dimensional parameter
*C*	compliance
*C_0_*	initial compliance
D	bending stiffness matrix
*D_ij_*	component of the bending stiffness matrix
*D_c_*	non-dimensional parameter
*E_1_*	longitudinal Young’s modulus
*E_2_*	transversal Young’s modulus
*E_f_*	flexural modulus
*G_12_*	shear modulus
*G_IC_*	critical strain energy release rate
*L*	specimen length
M	moment vector
N	force vector
*P*	applied load
*ν_12_*	Poisson’s ratio
*δ*	displacement
Δ	correction parameter
Γ	correction parameter
**List of acronyms**	
AE	acoustic emission
BE	bending-extension coupled specimen
BT	bending-twisting coupled specimen
CBBM	Compliance-Based Beam Method
DCB	Double Cantilever Beam
MCC	Modified Compliance Calibration
CCM	Compliance Calibration Method
MBT	Modified Beam Theory
SEM	scanning electron microscope
